# *Candida albicans* Meningoencephalitis After Vestibular Schwannoma Surgery: An Autopsy-Confirmed Case Report

**DOI:** 10.3390/diagnostics16020228

**Published:** 2026-01-11

**Authors:** Jessika Camatti, Matteo Tudini, Maria Paola Bonasoni, Anna Laura Santunione, Rossana Cecchi, Erjon Radheshi, Edoardo Carretto

**Affiliations:** 1 Department of Medicine and Surgery, University of Parma, Via Università 12, 43121 Parma, Italy; jessika.camatti@unipr.it; 2 Unit of Legal Medicine, Department of Medical and Surgical Sciences, University of Bologna, Via Irnerio 49, 40126 Bologna, Italy; matteotudini@hotmail.it; 3 Pathology Unit, Azienda USL-IRCCS di Reggio Emilia, Via Amendola 2, 42122 Reggio Emilia, Italy; 4 Unit of Legal Medicine, Department of Biomedical, Metabolic and Neural Sciences, University of Modena and Reggio Emilia, Via Campi 287, 41125 Modena, Italy; annalaura.santunione@unimore.it (A.L.S.); rossana.cecchi@unimore.it (R.C.); 5 Unit of Legal Medicine and Bioethics, Azienda USL-IRCCS di Reggio Emilia, Via Amendola 2, 42122 Reggio Emilia, Italy; erjon.radheshi@ausl.re.it; 6 Clinical Microbiology Laboratory, Azienda USL-IRCCS di Reggio Emilia, 42122 Reggio Emilia, Italy; edoardo.carretto@ausl.re.it; 7 U.O.C. Microbiologia e Virologia Azienda Socio Sanitaria Territoriale “Papa Giovanni XXIII”, 24127 Bergamo, Italy

**Keywords:** *Candida albicans*, meningoencephalitis, post-neurosurgical infection, cerebrospinal fluid, forensic pathology, forensic autopsy, forensic histopathology, β-d-glucan

## Abstract

**Background and Clinical Significance:** Cerebral candidiasis (*Candida albicans* meningoencephalitis) is a rare but severe central nervous system (CNS) infection, usually associated with neurosurgical procedures or indwelling devices. Diagnosis is challenging due to frequent negativity of cerebrospinal fluid (CSF) cultures, and mortality remains high despite antifungal therapy. **Case Presentation:** We describe a 64-year-old woman who underwent retrosigmoid resection of a left vestibular schwannoma. The early postoperative course was complicated by fever, neurological deterioration, and hydrocephalus requiring external CSF drainage. Multiple lumbar punctures revealed inflammatory CSF profiles but persistently negative cultures. One month post-surgery, intraoperative samples from mastoid repair material grew *Candida albicans*, prompting antifungal therapy. Despite treatment, the patient experienced fluctuating neurological status and required multiple external ventricular drains. Three months after surgery, she clinically deteriorated and died. Autopsy showed diffuse meningeal thickening and purulent exudates at the brain base and posterior fossa. Histopathology confirmed chronic lympho-histiocytic meningitis with necrotizing foci containing *Candida albicans*. **Conclusions:** This case underscores the diagnostic and therapeutic challenges of post-neurosurgical *Candida* CNS infections. Repeatedly negative CSF cultures delayed diagnosis, emphasizing the value of ancillary tests such as β-d-glucan and molecular assays. Even with antifungal therapy, prognosis is poor. Autopsy remains essential for uncovering fatal healthcare-associated fungal infections and informing clinical vigilance and medico-legal assessment.

## 1. Introduction

Fungal infections of the central nervous system (CNS) are uncommon but often devastating events, typically associated with immunosuppression, prolonged hospitalization, or neurosurgical interventions [1]. Among fungal pathogens, *Candida albicans* represents the most frequent commensal yeast of the human microbiota, yet cerebral candidiasis with CNS involvement is considered exceptional. Reported predisposing conditions include prolonged intensive care, the use of broad-spectrum antibiotics, and above all, neurosurgical procedures with implantation of foreign material or cerebrospinal fluid (CSF) diversion systems [2,3].

Over the past decades, the increasing use of neurosurgical devices such as external ventricular drains and ventriculo-peritoneal shunts has been recognized as a significant risk factor for CNS candidiasis [2,4]. The clinical presentation is usually nonspecific, ranging from persistent fever to neurological decline, seizures, or hydrocephalus, which overlap with bacterial or viral etiologies [3,5].

Diagnosis remains a major challenge. Standard CSF cultures, although considered the gold standard, frequently yield negative results, particularly in the early stages [6,7]. Repeated negative cultures may delay the initiation of targeted antifungal therapy, a factor strongly associated with unfavorable outcomes [3]. To improve sensitivity, ancillary tests such as CSF β-d-glucan and molecular methods have been proposed [8,9]. While these approaches have demonstrated encouraging results in research settings, their specificity, standardization, and routine applicability remain controversial, limiting widespread use [10,11].

Therapeutic strategies are guided by the Infectious Diseases Society of America (IDSA) recommendations, which suggest initial treatment with liposomal amphotericin B combined with flucytosine, followed by step-down therapy with fluconazole [12]. Nevertheless, clinical experience shows that prognosis remains poor even under guideline-based management, partly due to limited antifungal penetration into the CNS and the biofilm-forming properties of *Candida albicans* [13]. Biofilms not only impair drug penetration but also facilitate persistent infection and relapse. Consequently, removal of the infected device, whenever feasible, is regarded as a critical component of effective management and is consistently recommended as standard of care in CNS candidiasis [12].

The rarity of CNS candidiasis, the diagnostic uncertainty during life, and the consistently poor prognosis highlight the critical role of autopsy in documenting such cases. Post-mortem investigations not only provide definitive diagnosis and insight into the pathological processes but also contribute to clinical awareness and prevention strategies. Previous studies have demonstrated that autopsy continues to play a vital role in recognizing rare infections and clarifying unexpected causes of death, even in the modern era of advanced diagnostics [14].

Here, we describe a fatal case of cerebral candidiasis (*Candida albicans* meningoencephalitis) following vestibular schwannoma surgery. The case is of particular interest because the infection was difficult to diagnose as CSF cultures were repeatedly negative, and *Candida albicans* infection was microbiologically identified late in the disease course, after irreversible clinical deterioration, and was definitively confirmed at autopsy. By presenting this case, we aim to underscore the diagnostic challenges, therapeutic limitations, and forensic relevance of CNS candidiasis in the post-neurosurgical setting.

## 2. Case Report

A 64-year-old woman underwent retrosigmoid craniotomy for elective resection of a left vestibular schwannoma. The surgical procedure was uneventful, and immediate postoperative recovery was initially satisfactory. However, within the first postoperative week, the patient developed persistent fever (up to 38.5 °C), worsening headache, and fluctuating consciousness. Neurological examination revealed somnolence, disorientation, and mild dysarthria. Laboratory tests showed leukocytosis (14,200/µL), elevated C-reactive protein (CRP 12 mg/dL), and increased erythrocyte sedimentation rate (ESR). The patient had no history of malignancy, chronic immunosuppressive therapy, systemic autoimmune disease, or other conditions associated with immunodeficiency. No previous fungal infections were documented in the patient’s medical history.

Cranial computed tomography (CT) demonstrated progressive ventricular enlargement, consistent with obstructive hydrocephalus. No focal parenchymal lesions or abscess formations were identified on CT imaging. An external ventricular drain (EVD) was placed, resulting in transient clinical improvement. CSF analysis revealed pleocytosis (320 cells/µL, predominantly lymphocytes), elevated protein (310 mg/dL), and reduced glucose (23 mg/dL; blood glucose 95 mg/dL). Despite these inflammatory features, serial cerebrospinal fluid samples were routinely analyzed using standard bacterial and fungal cultures, which remained negative during the early phase of the disease. Empirical broad-spectrum antibiotics (ceftazidime, vancomycin, and later meropenem) were administered without clinical benefit.

During the following three weeks, the patient remained febrile with fluctuating neurological status, including episodes of confusion, generalized weakness, and declining Glasgow Coma Scale (GCS) scores. Magnetic Resonance Imaging (MRI) of the brain showed diffuse leptomeningeal enhancement, more pronounced at the skull base and posterior fossa, together with persistent ventricular dilation. These findings were compatible with meningitis of undetermined etiology. The enhancement pattern was diffuse and predominantly basal, without focal lesions or abscesses amenable to targeted biopsy.

The initial retrosigmoid procedure included mastoid reconstruction using non-autologous graft material to repair the skull base defect and prevent cerebrospinal fluid leakage. Approximately one month later, revision of the mastoid repair site was undertaken because of local inflammatory changes associated with cerebrospinal fluid leakage. No frank purulent material was observed at revision. Apart from this episode, the surgical wound remained clinically dry throughout the postoperative course.

At revision, the mastoid reconstruction site was reopened and revised. Intraoperative samples of the graft material were collected and sent for microbiological analysis, which yielded growth of *Candida albicans*, representing the first microbiological evidence of fungal infection. Antifungal therapy with liposomal amphotericin B (5 mg/kg/day) combined with flucytosine (100 mg/kg/day) was therefore initiated. Over the following weeks, treatment was transitioned to high-dose fluconazole (800 mg/day) because of cumulative toxicity.

Despite antifungal therapy, the clinical course remained unfavorable. Ventricular catheters were replaced for clinical reasons; however, microbiological examination of all removed devices was not systematically performed. Follow-up CSF analyses continued to show pleocytosis (ranging 150–400 cells/µL) and markedly elevated protein (>300 mg/dL). On two occasions, *Candida albicans* was isolated from CSF samples, confirming persistent infection. Neuroimaging performed during this period demonstrated diffuse basal meningeal thickening, persistent ventriculomegaly, and progressive involvement of the brainstem and hypothalamic regions.

The patient’s general condition deteriorated progressively. She developed severe encephalopathy, with GCS declining to 6, and required transfer to the intensive care unit. Supportive measures included mechanical ventilation, enteral nutrition, and management of electrolyte disturbances. Secondary complications arose, including ventilator-associated pneumonia, sepsis, and renal impairment related to prolonged antifungal therapy. Broad-spectrum antibiotics were reintroduced, but repeated blood cultures remained negative for bacteria.

Approximately three months after the initial neurosurgical procedure, the patient became comatose and developed multi-organ failure. Despite aggressive supportive therapy and continuation of antifungal treatment, she died.

### 2.1. Autopsy Findings

**Figure 1:**
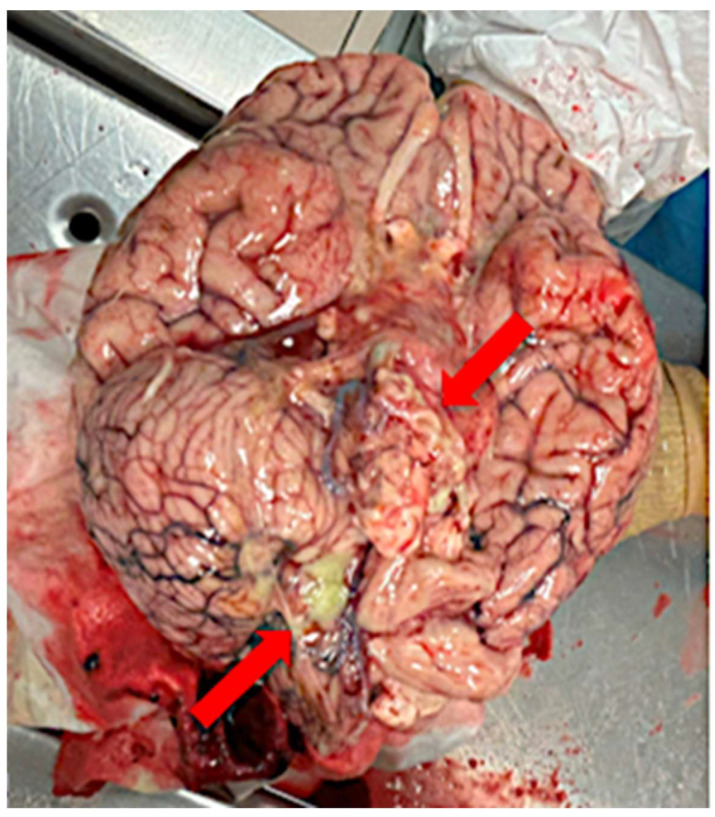
Autopsy findings at the foramen magnum showing yellow-green purulent exudate adherent to the basal meninges (red arrows).

A complete autopsy was performed. Externally, signs of neurosurgical intervention were evident at the left retrosigmoid region. On opening the cranial cavity, the basal meninges appeared diffusely thickened, opaque, and yellowish. At the level of the foramen magnum and posterior fossa, abundant yellow-green purulent material was noted (Figure 1). Microbiological swabs were taken and post-mortem microbiological cultures confirmed *Candida albicans*.

The cerebral hemispheres were edematous with flattened gyri. The brain was formalin fixed and examined after two weeks.

The lungs were congested, but they weighed within normal limits (right 700 g, left 500 g). Mild bilateral pleural effusions were also found (20 mL in each cavity). No other relevant anomalies were observed in the other organs.

**Figure 2:**
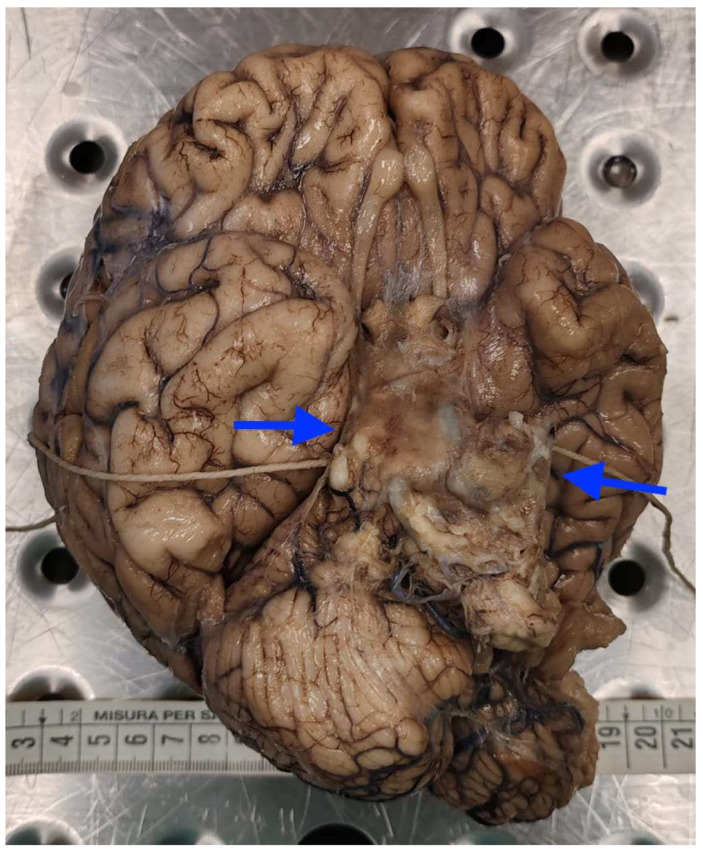
Brain candidiasis: the base of the brain (arrows) was wrapped up in thickened and opaque meninges.

**Figure 3:**
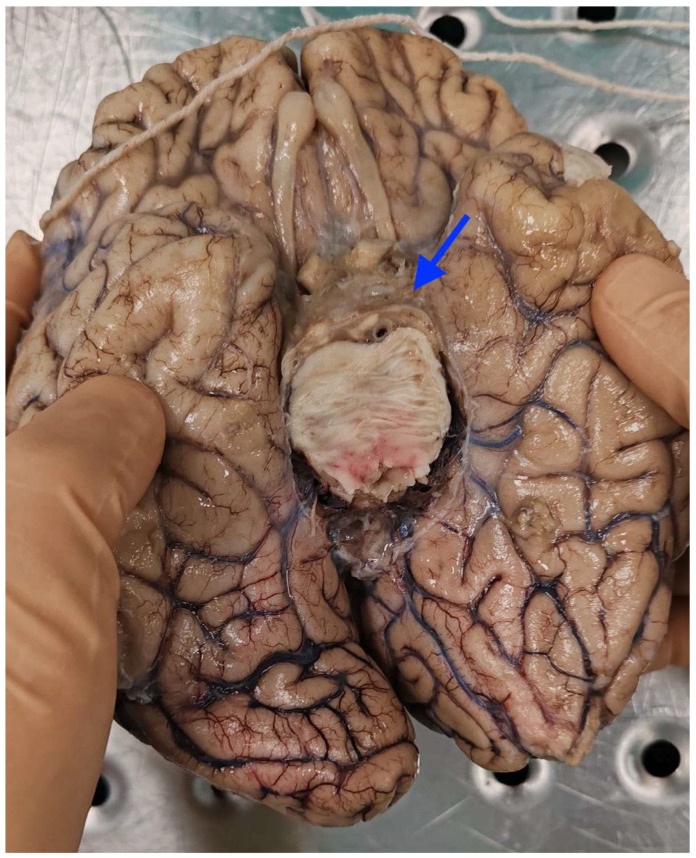
Brain candidiasis: the brain stem at the level of pons showed yellowish and particularly thickened meninges (arrow).

Neuropathological examination showed the typical macroscopic features of “cerebral candidiasis”. The brain was covered by meninges diffusely opaque, especially in the parietal and temporal regions. At the level of the cranial base, in the pituitary region, the meninges were thickened, yellowish, and opaque. The meninges were also opaque and thickened at the level of the brainstem (Figure 2 and Figure 3).

The brain stem was fully included and multiple samples were included from both emispheres, deep gray nulcei, and the cerebellum.

Histologically, the brain showed the typical “brain candidiasis”.

**Figure 4:**
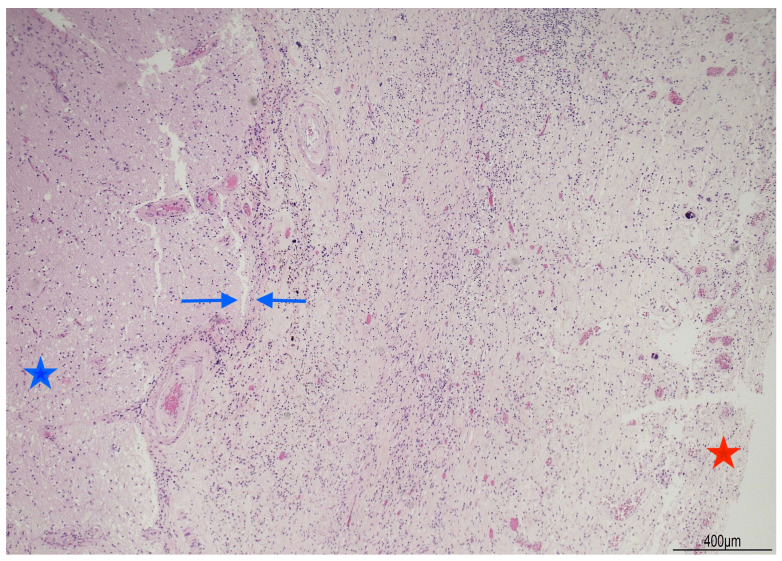
Brain candidiasis with chronic lympho-histiocytic meningitis: at brain stem level (blue star) meninges (red star) were markedly thickened showing a diffuse fibrous proliferation mixed with lymphocytes and histiocytes. The blue arrows indicate the edge between the brain stem and the meninges (Hematoxylin and Eosin, 4HPF).

**Figure 5:**
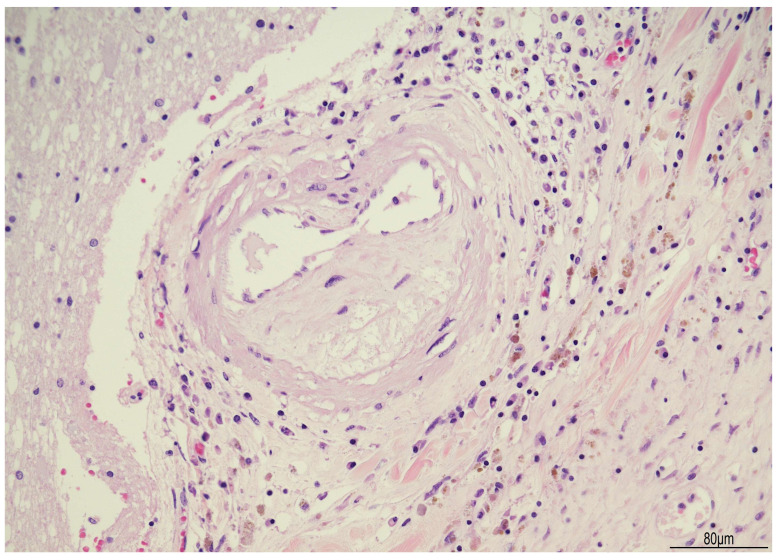
Brain candidiasis with lymphoplasmacytic vasculitis: most of the vessels were surrounded by lymphocytes and plasma cells. Vascular atherosclerosis was widespread in the encephalon, as seen as an example in this picture. Hemosiderin deposition was also a common finding (brown granules) (Hematoxylin and Eosin, 20HPF).

Brain stem histopathology displayed chronic lympho-histiocytic meningitis with plasma cells and some multinucleated giant cells; multifocal necrotizing, abscess-forming areas with granulocytes; lymphoplasmacytic vasculitis; multifocal calcifications and circumscribed deposits of hemosiderin (Figure 4 and Figure 5).

**Figure 6:**
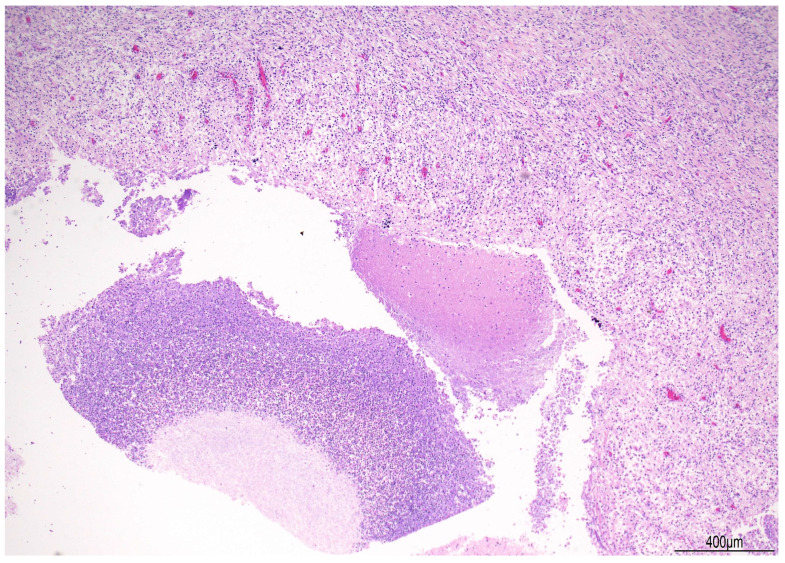
Meningeal brain candidiasis at the level of pituitary stalk: abscess formation with central necrosis surrounded by chronic lympho-histiocytic meningitis (Hematoxylin and Eosin, 4HPF).

**Figure 7:**
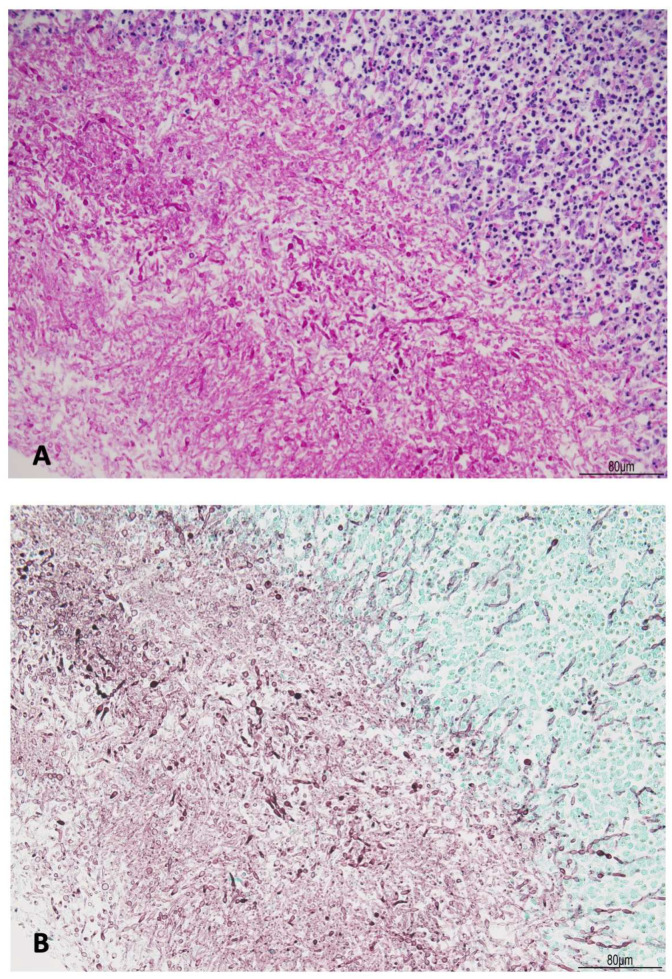
Abscess formation in meningeal brain candidiasis (pituitary stalk): budding yeast forms and pseudohyphae of *Candida albicans* were highlighted by periodic acid-Schiff diastase (PAS-D, (**A**)) and Gomori methenamine silver (GMS, (**B**)) (Hematoxylin and Eosin, 20HPF).

Chronic lympho-histiocytic meningitis with plasma cells and lymphoplasmacytic vasculitis were also found in the optic chiasm, in the hypothalamus, within the pituitary stalk, in the hippocampal region, and in the posterior meningeal falx. Within the pituitary stalk, within meninges, a central core full of budding yeast forms and pseudohyphae compatible with *Candida albicans* were observed, and confirmed by histochemistry for Gomori methenamine silver (GMS) and periodic acid-Schiff diastase (PAS-D) (Figure 6 and Figure 7).

Lymphocytic ventriculitis was found in the thalamic region, basal ganglia, and along the Silvian aqueduct. In the latter, in the lumen, at the mesencephalic region, a few hyphae and fungal spores compatible with *Candida albicans* were detected.

Lymphocytic meningitis, without plasma cells, was more prominent in the frontal, parietal, temporal, and occipital regions. Vascular atherosclerosis was widespread in the brain and cerebellum.

Cerebral parenchyma was overall preserved with no abscess formation. Focal areas of leukomalacia and gliosis were found close to the most involved meninges.

Additional autopsy findings included acute bronchopneumonia and pulmonary edema, consistent with terminal complications. Other major organs showed non-specific changes in critical illness. No evidence of systemic immunodeficiency, malignancy, or chronic corticosteroid therapy was found at autopsy, supporting the interpretation that neurosurgical manipulation and CSF device management represented the main predisposing factors.

### 2.2. Summary of Clinical Course

To illustrate the patient’s progression, the principal milestones of clinical presentation, microbiology, imaging, and therapeutic interventions are summarized in Table 1.

## 3. Discussion

### 3.1. Overview of the Case

The present case illustrates a rare and fatal cerebral candidiasis (*Candida albicans* meningoencephalitis) following vestibular schwannoma resection. Despite repeated CSF analyses, prolonged antibacterial therapy, and initiation of antifungal treatment once the pathogen was identified, the patient experienced progressive neurological decline and died three months after the neurosurgical procedure. This clinical trajectory underscores the formidable diagnostic and therapeutic challenges posed by CNS candidiasis.

### 3.2. Epidemiology and Risk Factors

CNS candidiasis is an uncommon condition, reported mainly in immunocompromised individuals, premature neonates, and patients subjected to neurosurgical interventions [15]. Neurosurgical procedures are now considered a major predisposing factor, particularly when foreign material is implanted or when repeated CSF drainage is required [1,2,3,4]. In our case, no intraoperative contamination or surgical complication was documented. However, a plausible mechanism of infection involves secondary fungal colonization facilitated by skull base surgery and mastoid reconstruction, which may have created a favorable anatomical and biological environment for microbial seeding. In addition, prolonged CSF diversion with external ventricular drainage likely promoted biofilm formation on indwelling devices, enabling persistent fungal colonization and subsequent invasion of the skull base and basal meninges.

A comprehensive review of invasive candidiasis reported mortality rates approaching 70% despite antifungal therapy [15]. Sánchez-Portocarrero et al. noted neurocandidiasis often in adults with neurosurgical history or CSF device placement as predisposing conditions, among other risk factors [16]. This emphasizes that, although rare, *Candida* infections of the CNS should always be considered in neurosurgical patients with unexplained fever, hydrocephalus, or culture-negative meningitis.

### 3.3. Diagnostic Challenges

Diagnosis of CNS candidiasis is notoriously difficult. Classical CSF abnormalities—pleocytosis, elevated protein, and hypoglycorrhachia—are nonspecific and overlap with bacterial and viral meningitis [1,3,5]. Moreover, CSF cultures, although considered the gold standard, are frequently negative, especially in the early course of disease [4,6,7]. In our patient, repeated cultures were sterile for several weeks, leading to prolonged empirical antibacterial therapy and delayed initiation of antifungal treatment. Notably, the absence of frank purulence at revision surgery further contributed to the diagnostic uncertainty.

Ancillary tests have been investigated to overcome these limitations. CSF β-d-glucan has been reported as highly sensitive for fungal meningitis, with detection rates above 80% in some series [7,9,10]. However, specificity remains an issue, and false positives can occur in the context of bacterial infection, hemodialysis, or intravenous immunoglobulin therapy [9,10]. Molecular assays, including broad-range fungal PCR and next-generation sequencing, have also shown promise in rapidly identifying fungal DNA directly from CSF [6,8,11]. Yet these methods are not universally available, and issues of standardization, cost, and turnaround time limit their routine application [6,8,11].

In the present case, β-d-glucan and molecular tests were not performed, which reflects real-world limitations but also highlights an area for improvement. Earlier use of such tools might have facilitated timelier antifungal therapy [3,4,6].

### 3.4. Therapeutic Considerations

Once *Candida albicans* was identified, the patient received amphotericin B and flucytosine, followed by fluconazole, in line with the Infectious Diseases Society of America (IDSA) guidelines [12]. While a more aggressive diagnostic and therapeutic approach, including intrathecal antifungal therapy or additional invasive procedures, may be justified in selected critically ill patients, these options were not pursued in the present case because of the absence of focal lesions amenable to biopsy, the high procedural risk, and the patient’s rapidly progressive clinical deterioration.

Despite adherence to recommended therapy, the infection progressed, leading to death. This outcome is consistent with previous reports, which document high mortality rates even in patients treated with appropriate antifungal regimens [1,3,4,13].

Several factors may contribute to treatment failure. The penetration of amphotericin B and flucytosine into the CSF is variable, and fluconazole, although more readily diffusible, may be less effective in eradicating established infections [11,12]. Furthermore, *Candida* species are capable of forming biofilms on indwelling devices, including ventricular drains, which markedly reduces antifungal susceptibility [1,17]. Device-associated infections therefore often require not only antifungal therapy but also removal or replacement of infected hardware—a strategy that was repeatedly attempted in our patient, yet infection persisted.

Alternative antifungals such as voriconazole or echinocandins have limited evidence for efficacy in CNS candidiasis, primarily due to poor CSF penetration [1,2,13]. While echinocandins remain first-line therapy for many invasive *Candida* infections, their effectiveness in sanctuary sites such as the CNS is unproven [13]. Thus, despite modern antifungal options, CNS candidiasis remains a condition with dismal prognosis.

### 3.5. Role of Autopsy

In this case, autopsy was essential in confirming the diagnosis and delineating the full extent of the infection. Macroscopically, thickened basal meninges with purulent exudates were observed, while histopathology confirmed diffuse fungal infiltration with yeast forms and pseudohyphae. Without autopsy, the diagnosis would have remained presumptive, and the true contribution of *Candida albicans* to the fatal outcome could have been questioned.

This finding reinforces the enduring value of autopsy in clarifying diagnostically elusive and unexpected infectious deaths, even in the era of advanced imaging and molecular diagnostics. Similar considerations on the diagnostic role of autopsy in unexpected and fulminant infectious deaths have been reported by Di Fazio et al. [18], further supporting the relevance of post-mortem investigations in complex clinical scenarios.

### 3.6. Mini-Review of Published Cases

To contextualize our findings, we reviewed published literature on CNS *Candida* infections with emphasis on post-neurosurgical settings. A synthesis is presented in Table 2.

Overall, the reviewed literature confirms CNS candidiasis as a rare but severe healthcare-associated infection, most frequently occurring in patients with prior neurosurgical procedures, implanted foreign material, or prolonged cerebrospinal fluid diversion, even in the absence of overt immunosuppression [2,3,4,21].

A consistent feature across the literature is the marked diagnostic difficulty. Cerebrospinal fluid findings are often nonspecific, and conventional fungal cultures show limited sensitivity, frequently remaining negative despite ongoing infection [2,3,20]. As a consequence, diagnosis is often delayed, with *Candida* identified only after repeated CSF sampling, removal of infected devices, or invasive diagnostic procedures. Several reports describe prolonged empiric antibacterial or anti-tubercular therapy before initiation of appropriate antifungal treatment [6,21].

Device-associated infection and biofilm formation represent central pathogenic mechanisms repeatedly emphasized in published cases. External ventricular drains, ventriculoperitoneal shunts, lumbar drains, and other intracranial foreign materials are frequently implicated as reservoirs for persistent fungal infection, often necessitating device removal or replacement [2,3,7]. Despite antifungal therapy, reported mortality remains high, and survivors frequently experience severe or permanent neurological sequelae [2,21].

More recent publications have highlighted the potential role of advanced diagnostic tools as adjuncts to conventional microbiology. Cerebrospinal fluid β-d-glucan testing has been proposed as a sensitive biomarker for fungal meningitis, including *Candida* infections, particularly in cases with persistently negative cultures [7,20]. In addition, molecular approaches such as pan-fungal PCR and metagenomic next-generation sequencing have demonstrated diagnostic value in selected cases of chronic or culture-negative *Candida* meningitis, although their availability remains limited and experience is still evolving [6].

Compared with previously published cases, the present report is distinguished by the availability of complete autopsy findings, which allowed definitive confirmation of the diagnosis and precise delineation of the extent of meningeal and parenchymal involvement. Such comprehensive clinicopathological correlation is rarely documented in the literature and further highlights both the limitations of ante-mortem diagnostic strategies and the continued relevance of autopsy in clarifying fatal healthcare-associated fungal infections.

### 3.7. Forensic Perspective and Ancillary Methods

From a forensic and medico-legal standpoint, this case reinforces the role of autopsy as a tool for both diagnostic confirmation and broader scientific contribution. In forensic medicine, ancillary techniques such as immunohistochemistry, biomarker quantification, and post-mortem imaging have expanded the diagnostic spectrum [22,23,24,25,26,27,28]. These approaches parallel the application of PAS-D, GMS staining, and microbiological cultures in the present case, highlighting the common methodological ground between forensic and clinical pathology.

Moreover, autopsy findings provide valuable epidemiological data and inform preventive strategies. Documenting healthcare-associated infections (HAIs), such as post-neurosurgical CNS candidiasis, has implications not only for individual patient care but also for institutional risk management and public health surveillance. Forensic case series, such as fatal infections [14], exemplify how autopsy-based research can generate insights that extend beyond individual cases. Similarly, reporting rare fungal infections contributes to raising clinical awareness and improving diagnostic vigilance in neurosurgical practice.

### 3.8. Medico-Legal Implications

Healthcare-associated fungal infections pose complex medico-legal challenges. While not all cases are preventable, delayed diagnosis or failure to consider fungal pathogens may raise questions of clinical responsibility. In the present case, despite appropriate management once *Candida* was identified, the long diagnostic delay contributed to therapeutic failure. This underlines the importance of maintaining broad differential diagnoses in postoperative meningitis, especially when cultures remain negative and the clinical course is atypical. Autopsy, by establishing the definitive cause of death, ensures transparency and accountability, while also guiding preventive strategies to minimize future risk.

### 3.9. Strengths and Limitations of the Case

The strength of this case lies in the comprehensive clinical documentation combined with definitive autopsy confirmation, which is rarely available in modern case series. Histopathological and microbiological findings were concordant, leaving no doubt about the diagnosis.

Several limitations should also be acknowledged. These include the absence of advanced diagnostic assays during life, such as CSF β-d-glucan testing or PCR-based fungal detection, which were not available in routine clinical practice at the time of the patient’s hospitalization, as well as the lack of antifungal susceptibility testing. Nonetheless, these limitations reflect real-world practice, where such tests are not always accessible. Additional limitations include the inability to pursue intrathecal antifungal therapy or further surgical revisions because of the patient’s rapid clinical deterioration and the unfavorable risk–benefit profile of these interventions. Moreover, microbiological analysis of removed ventricular catheters was not systematically performed and might have facilitated earlier pathogen identification.

## 4. Conclusions

This case highlights several clinically relevant points. First, fungal etiology should be considered early in post-neurosurgical meningitis, particularly in the presence of persistently negative CSF cultures. Second, standard microbiological cultures have limited sensitivity in CNS candidiasis and may delay targeted antifungal therapy. Third, careful management and timely removal or replacement of indwelling CSF devices are crucial, as biofilm-associated infections are difficult to eradicate with antifungal treatment alone. Finally, autopsy remains an indispensable tool for confirming elusive diagnoses, clarifying unexpected deaths, and improving clinical awareness of rare but fatal healthcare-associated infections.

## Figures and Tables

**Table 1:** Summary of clinical course, diagnostic findings, and treatments.

Postoperative Time	Clinical Course	CSF/Microbiology	Imaging	Treatment
Early postoperative days	Fever, neurological deterioration, hydrocephalus requiring external ventricular drain	CSF: pleocytosis, high protein; cultures negative	Ventricular enlargement (hydrocephalus)	Empirical broad-spectrum antibiotics
~1 month after surgery	Fluctuating consciousness, persistent fever	Intraoperative mastoid graft cultures: *Candida albicans*	Basal meningeal enhancement on MRI	Liposomal amphotericin B + flucytosine, later switched to fluconazole
Weeks 5–10	Recurrent hydrocephalus, need for repeated drainage procedures; partial, transient improvement	CSF: inflammatory profile; intermittent *Candida albicans* isolation	Persistent ventricular dilation, basal meningeal enhancement	Continued antifungal therapy
~3 months after surgery	Progressive neurological decline, coma, death	Autopsy: PAS-D and GMS stains positive for fungal elements (*Candida albicans*)	–	Supportive intensive care until death

**Table 2:** Published reports on *Candida* CNS infections in neurosurgical or device-related settings.

Study (Year)	Design/Cohort	Setting & Risk Factors	Diagnostic Confirmation	Ancillary Tests	Treatment Notes	Outcome/Key Message
Nguyen & Yu (1995) [19]	Case series (*n* = 3)	Neurosurgical patients	CSF/tissue culture	–	Amphotericin B ± flucytosine	Highlighted CNS candidiasis as an emerging complication in neurosurgery; poor prognosis.
Sánchez-Portocarrero et al. (2000) [16]	Review	Mixed: meningitis & abscesses; device-related	Literature-based	–	Amphotericin B + flucytosine	Classic overview stressing device role and high mortality.
O’Brien et al. (2011) [2]	12-year institutional review	Post-neurosurgical; foreign body implants	Culture/histology	–	Device removal + antifungals	Confirmed strong link with foreign material; guarded prognosis.
Chen et al. (2020) [4]	Series (*n* = 9)	8/9 device-associated (VPS, LPS, EVD)	Serial CSF cultures	–	Fluconazole/voriconazole; hardware removal	Mortality 11.1%; survivors with severe sequelae.
Chen et al. (2021) [3]	Series + review	Post-neurosurgical; prior bacterial CNS infection common	CSF culture (delayed positivity)	–	Amphotericin B + azoles; device strategy	Emphasized diagnostic delays.
Lyons et al. (2013, 2015) [7,20]	Case reports	Meningitis, some iatrogenic	Culture/clinical	CSF β-d-glucan	Antifungals per case	β-d-glucan useful when cultures negative.
Bigot et al. (2023) [21]	Multicenter retrospective	Non-cryptococcal fungal CNS infections	Composite reference	CSF β-d-glucan	–	BDG promising but not standardized.
Kuenzli et al. (2024) [6]	Case report	Shunt-associated chronic meningitis	mNGS on CSF	mNGS	Targeted azoles	Showed diagnostic delay, value of molecular methods.

## Data Availability

The data presented in this study are available on request from the corresponding author.

## Abbreviations

The following abbreviations are used in this manuscript:

CSF	Cerebrospinal Fluid
CNS	Central Nervous System
CT	Computed Tomography
EVD	External Ventricular Drain
GCS	Glasgow Coma Scale
MRI	Magnetic Resonance Imaging
PAS-D	Periodic Acid–Schiff with Diastase
HAIs	Healthcare-Associated Infections
